# Specificity and overlap in gene segment-defined antibody repertoires

**DOI:** 10.1186/1471-2164-6-148

**Published:** 2005-10-28

**Authors:** Ramy A Arnaout

**Affiliations:** 1 Department of Pathology, Brigham and Women's Hospital, Boston, MA 02115 USA; 2 The Broad Institute, Cambridge, MA 02141 USA; 3 Program for Evolutionary Dynamics, Harvard University, Cambridge, MA 02138 USA

## Abstract

**Background:**

To date several studies have sought to catalog the full suite of antibodies that humans naturally produce against single antigens or other specificities (repertoire). Here we analyze the properties of all sequenced repertoires in order to better understand the specificity of antibody responses. Specifically, we ask whether the large-scale sequencing of antibody repertoires might provide a diagnostic tool for detecting antigen exposure. We do this by examining the overlap in V_*H*_-, D-, and J_*H*_- segment usage among sequenced repertoires.

**Results:**

We find that repertoire overlap in V_*H*_-, D-, and J_*H*_-segment use is least for V_*H *_segments and greatest for J_*H *_segments, consistent with there being more V_*H *_than J_*H *_segments in the human genome. We find that for any two antigens chosen at random, chances are 90 percent that their repertoires' V_*H *_segments will overlap by less than half, and 98 percent that their VDJ_*H *_combinations will overlap by ≤10 percent. We ran computer simulations to test whether enrichment for specific VDJ_*H *_combinations could be detected in "antigen-exposed" populations, and found that enrichment is detectable with moderate-to-high sensitivity and high specificity, even when some VDJ_*H *_combinations are not represented at all in some test sets.

**Conclusion:**

Thus, as large-scale sequencing becomes cost-effective for clinical testing, we suggest that sequencing an individual's expressed antibody repertoire has the potential to become a useful diagnostic modality.

## Background

The antigen-binding variable regions of antibody molecules draw combinatorially from a set of somatically encoded V, D, and J gene segments [1]. Mathematically, this strategy allows for ~6,000 possible heavy chain (subscript _*H*_) and ~300 possible light chain (subscript _*L*_) V(D)J combinations, for a total of ~1.8 million possible heavy-and-light chain pairings [2,3].

Much work in immunology and structural biology has gone into studying how antibody sequence and structure affect antigen specificity [1]. In each antibody, contact with the antigen is made by six short regions, three on each heavy and light chain. These are known as the complementarity-determining regions (CDRs). CDR1 and CDR2 lie entirely within the V segment, while CDR3 spans the D segment and flanking parts of V and J (in heavy chain; in light chain, which lacks a D segment, CDR3 spans the V-J junction). In general, heavy chain contributes more than light chain to antigen binding and specificity, and CDR3 contributes more than CDR1 and CDR2 [4]. Hence heavy chain VDJ (VDJ_*H*_) segment usage is a major determinant of antigen specificity.

There are other determinants. The part of an antigen that an antibody binds is called an epitope; the part of an antibody that an epitope binds is called a paratope. Single antigens may have multiple epitopes, and single antibodies may have multiple paratopes [5,6]. Moreover, nontemplated nucleotide insertions and deletions at gene segment junctions, together with CDR hypermutation, expand antibody diversity and antigen binding possibilities far beyond what is available through V(D)J combinatorics alone [1]. Hence V(D)J segment choice and sequence-level modification provide coarse- and fine-tuning, respectively, for antigen specificity, but different V(D)J and sequence combinations may well bind the same antigen.

These considerations and substantial experimental data (summarized in [4]) argue against a strict one-to-one relationship between antibody sequence and antigen specificity. However, they do suggest the possibility that antigens may have signature antibody repertoires. Here a repertoire is defined as a set of antibodies, defined by gene segment usage, that is produced in a population of people against a given specificity. A specificity comprises a single epitope, a set of epitopes on a single antigen, or a set of antigens.

To date several studies have addressed this idea in particular instances by sequencing antibodies specific for particular antigens. In one such study, circulating B cells from seven infants vaccinated against *Hemophilus influenzae *type b (Hib) were affinity enriched aganst Hib capsular polysaccharide (PS); rearranged V(D)J heavy and light chain gene libraries were then constructed and screened for Hib PS-specific antibodies [7]. The antibodies recovered all used the same V_*H *_segment (V_*H*_3–23) and only two J_*H *_and two V_*L *_and J_*L *_segments, consistent with previous studies [8,9]. This is consistent with the pattern seen in natural antibody populations, allowing consideration of data from this *in vitro *"scrambling" approach.

Repertoires against other antigens have also been shown to have restricted segment usage, although the degree and pattern of restriction vary. For example, using a technique similar to that described for Hib PS, the repertoire against *Streptococcus pneumoniae *serotype 23F PS was found to be dominated by four V_*H *_segments, which account for 90 percent of the repertoire's observed V_*H *_diversity; four J_*H *_segments (93% of J_*H *_diversity); and two V_*L*_-kappa segments (93%) [10]. For comparison, the repertoire against *S. pneumoniae *serotype 6B PS was found to be dominated by three V_*H *_segments (93%) and three J_*H *_segments (98%), but was found to lack strong V_*L*_-kappa restriction (90% in six segments) [11]. Association patterns among segments and chains were also found to vary.

In all, repertoires for over a dozen antigens have been studied individually, with various aims and to various extents, mainly through enrichment and cloning or through screening of phage-display libraries [7,10-14]. The aim of the present study is to analyze these repertoires as a group in order to better understand the specificity of antibody responses. The practical goal is to explore the possibility that in the future, large-scale sequencing of antibodies in an individual may be used as a fingerprint, or "pan-scan," of that person's antigen exposure.

## Results

We analyzed VDJ_*H *_segment usage for the 16 best-represented natural human repertoires in the IMGT database (see Methods). These comprised 292 antibody sequences (mean, 18 sequences per repertoire; range, 8–41). Six repertoires were directed against infectious agents, while 10 were directed against autoimmune agents (Table 1 and Additional File 1).

**Table 1:** Repertoire composition

specificity	sequences	V_*H *_genes	D genes	J_*H *_genes	VDJ_*H *_combos
*E. histolytica*	9	7	5	2	7
HBsAg (HBV)	12	9	8	3	11
PS (*S. pneumo *23F)	23	7	10	4	15
gp120 (HIV)	26	10	16	6	24
PS (*S. pneumo *6B)	41	5	10	3	11
dsDNA (human)	8	7	6	4	8
MAG (human)	9	7	7	4	9
PL (human)	9	8	8	4	9
Fab (human)	11	9	7	3	10
factor VIII (human)	19	3	5	3	6
cardiolipin (human)	12	7	7	3	10
gpIIb/IIIa (human)	14	12	10	3	14
myosin (human)	14	12	10	5	14
RhD (human)	22	9	14	5	20
DNA (human)	22	12	13	4	20
TPO (human)	41	6	11	4	16

total	292	36	21	6	192

Number of sequences, gene segments, and gene segment combinations in the repertoires of all specificities in the data set. Specificities associated with infectious agents listed first; species names within parentheses (where necessary). Abbreviations: *E. histolytica*, *Entameba histolytica*; HBV, hepatitis B virus; PS, polysaccharide; *S. pneumo*, *Streptococcus pneumoniae*; ds, double-stranded; MAG, myelin-associated glycoprotein; PL, phospholipid; TPO, thyroid peroxidase.

### Gene segment usage patterns

Genome-level diversity was well represented among the repertoires as a group. All but one (V_*H*_7) of the V_*H *_and D gene segment families were represented, and the majority of individual V_*H *_(78%), D (91%), and J_*H *_(100%) gene segments appeared in at least one sequence. V_*H *_and D gene families were represented about as often as in a previous study of healthy individuals [15] (*p *= 0.01 and 0.13, *R*^2 ^= 0.78 and 0.96 for V_*H *_and D families, respectively), as were individual J_*H *_gene segments (*p *= 0.004, *R*^2 ^= 0.94). However, individual V_*H *_gene segments were used more variably (*p *= 0.90, *R*^2 ^= 0.25).

These observations are consistent with there being more than one VDJ_*H *_combination used in antibodies with a given specificity (see below). They also suggest either that our set of repertoires is a good representation of at least the kinds [16] of antigen or antigen patterns encountered naturally, or conversely that B cell populations of the healthy individuals sampled in the previous study [15] comprise clones expanded against specificities similar to the ones included in our present analysis. These possibilities are not mutually exclusive.

**Figure 1:**
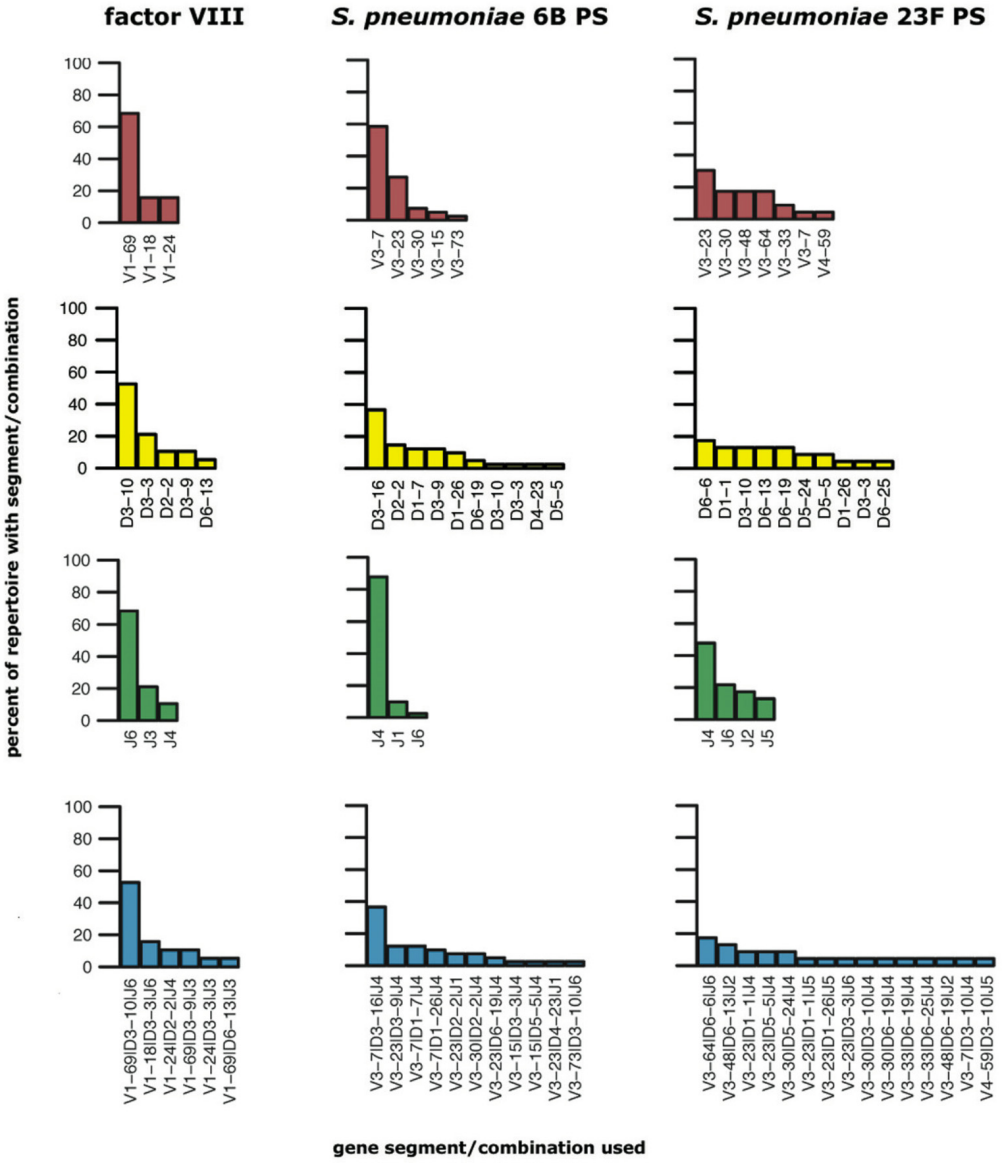
**Gene segment use for representative repertoires**. Repertoires for three specificities are shown: human coagulation factor VIII, *Streptococcus pneumoniae *serotype 6B capsular polysaccharide (PS), and *S. pneumoniae *ser. 23F PS. Each histogram shows the frequency distribution of V_*H *_gene segments, D segments, J_*H *_segments, and VDJ_*H *_combinations. More peaked distributions indicate that the repertoire is V_*H*_, D, J_*H*_, or VDJ_*H *_restricted. For example, the *S. pneumoniae *ser. 6B repertoire is 80% restricted to J_*H *_gene segment J_*H*_4.

Figure 1 shows V_*H*_, D, and J_*H *_segment usage and VDJ_*H *_combination usage patterns for the repertoires of representative specificities. Some repertoires were peaked and narrow, suggesting few epitopes or immunodominance among the epitopes in their specificities, or little diversity among individuals for these specificities ("public" or "semi-public" repertoires; see Discussion). Other repertoires were flat and broad, suggesting many epitopes or codominance among the epitopes in their specificities, or greater diversity among individuals for these specificities. Details of V_*H*_, D, and J_*H *_segment usage for particular specificities have been discussed elsewhere (see references for specific sequences in IMGT, Table 1, and Additional File 1).

The data did not allow conclusive generalization about whether or not, for a given repertoire, V_*H*_, D, and J_*H *_segments are combined randomly or with some bias. This is because the number of antibodies sequenced in a given repertoire was small (8–41 sequences) relative to the number of VDJ_*H *_combinations that could in principle be constructed from the V_*H*_, D, and J_*H *_segments that appeared in that repertoire (~50–1,000 possibilities).

For only one of the 16 repertoires – the repertoire for thyroid peroxidase – was there a tight, statistically significant correlation between the observed frequencies of VDJ_*H *_combinations and the frequencies that would be expected if segments were combined at random (*p *< 0.01; *R*^2 ^= 0.85). The repertoire for *S. pneumoniae *strain 6B polysaccharide also showed a tight correlation, but this correlation fell short of statistical significance (*p *= 0.10; *R*^2 ^= 0.95). No tight, statistically significant correlation was observed for any other repertoire. These findings are consistent with the conclusion that V_*H*_, D, and J_*H *_segments are not joined at random in at least 14 of these 16 repertoires, but more sequencing is needed to settle this issue.

### Overlap in gene segment usage

**Figure 2:**
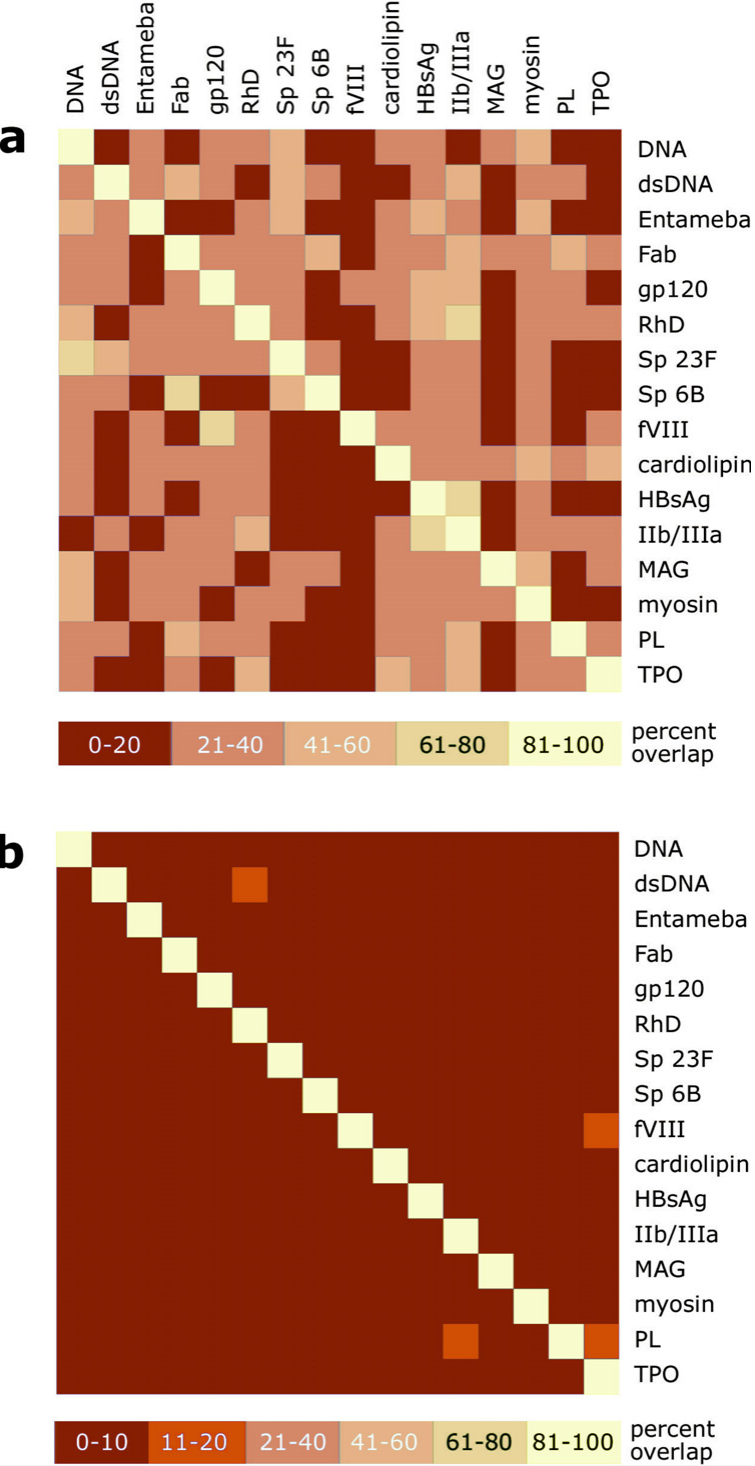
**Overlap in segment use among repertoires**. Overlap in (a) V_*H *_gene segment and (b) VDJ_*H *_use among all repertoires. The percent overlap is grayscale-coded according to the key below each plot. In (b), the range at the lower end of the scale is expanded in order to show the four pairs with 11–20 percent overlap (see text). Abbreviations: ds-DNA, double-stranded DNA; gp120, HIV-1 gp120; Sp, *Streptococcus pneumoniae *serotype; fVIII, clotting factor VIII; HBsAg, HBV surface antigen; IIb/IIIa, glycoprotein IIb/IIIa; MAG, myelin-associated glycoprotein; PL, phospholipid; TPO, thyroid peroxidase. Species of origin are as in Table 1.

From a practical perspective, for repertoires to serve as signatures for particular specificities, the overlap in gene segments or in V(D)J combinations among different repertoires must be low. To estimate this overlap quantitatively, we calculated the percent overlap between each pair of specificities in the data set (Fig. 2).

We found that for any two specificities picked at random from our set, the probability was 90 percent that their repertoires' V_*H *_gene segment usage overlapped by half or less (Fig. 2a, red tones). Adding D and J_*H *_segment information decreased the overlap markedly: of the 240 pairwise comparisons between different specificities in our data set, only four (1.7%) showed more than 10 percent overlap: between dsDNA and RhD (12%), thyroid peroxidase (TPO) and factor VIII (16%), TPO and phospholipid (11%), and phospholipid and integrin gpIIb/IIIa (11%) – all autoimmune specificities. Although not random in segment usage, autoimmune antibodies may share common features that result from impaired negative selection. Overall, for any two specificities chosen at random, the probability was 98.3 percent that their repertoires' VDJ_*H *_*combinations *overlapped by 10 percent or less (Fig. 2b).

Given the large number of possible VDJ_*H *_combinations (~6,000) and the relatively small size of the data set (292 sequences), it is reasonable to ask whether or not such a small amount of overlap is likely to occur by chance. Probability calculations show that it is not. The two most common human haplotypes allow a maximum of 5,244 and 6,348 possible functional VDJ_*H *_combinations, respectively; the probability that the small amount of overlap observed in our data should arise by chance is *p *= 0.004 (0.4%) and 0.011 (1.1%) for these two haplotypes, respectively (see Methods). Note that nonrandom association among V_*H*_, D, and J_*H *_segments means that only a fraction of these 5,244 or 6,348 possible combinations are actually observed. The smaller the number of combinations, the higher the probability that repertoires will overlap by chance. Hence the small amount of overlap observed in the data is even less likely to be the result of chance than these calculations suggest. The probabilities are therefore upper limits.

If the specificities analyzed in this study are indeed representative of the specificities to which human beings are exposed (see above), this finding suggests that VDJ_*H*_-defined sequences may be able to distinguish dependably among a wide variety of specificities.

### Simulating detection

For repertoires to be of practical use, it must be possible to detect when certain VDJ_*H *_combinations are present at a higher-than-background frequency. This may indicate, for example, prior or ongoing exposure to an infectious agent or the presence of a response to a vaccine [17]. Ideally detection should be possible even when this frequency is barely above background – that is, when the signal-to-noise ratio is low.

To test whether enrichment might be detectable, we ran computer simulations for each specificity. These were done briefly as follows (for details, see Methods). For each specificity, we assembled several sets of sequences that were each enriched for sequences of that specificity's repertoire. (The analogy is that each set of sequences corresponds to what might be obtained from a blood sample of an individual known to have a clinical history of that specificity.) The collection of these sets was our "reference collection" for the test (medically, the gold standard). The strategy was to see if test sets could be assigned as exposed or unexposed by comparing their patterns of VDJ_*H *_combinations to the ones from the reference collection. If antibodies in a test set had a similar pattern and prevalence of VDJ_*H *_combinations as those in the reference collection, the test set was assigned as "exposed." If the patterns were dissimilar, the test set was assigned as "unexposed." Assignment was performed with the aid of a computerized algorithm (see Methods).

We tested this approach for each specificity by seeing how well exposed and unexposed sets could be assigned. In clinical infections, B cells specific for an infectious agent rarely exceed 5–10 percent of the total B cell population. Therefore, as a conservative test, the sets in the reference collection had only 1–2.5 percent of their VDJ_*H *_combinations purposely drawn from the repertoire for the given specificity. For example, in testing for exposure to HIV gp120, of 1,000 VDJ_*H *_combinations determined for a set in the reference collection, only 10–25 would be guaranteed to be combinations that appeared in the HIV gp120 repertoire; the rest would be from the repertoires of *S. pneumoniae *serotype 6B PS, double-stranded (ds) DNA, and the other 14 specificities. Note that in this approach not all combinations are guaranteed to appear in any one set; however, the more frequently a combination appears in the repertoire – the higher its prevalence – the more likely (and more often) it is to appear in a given set. Also, the larger the reference collection, the more likely that less prevalent combinations will also appear in at least one set.

**Figure 3:**
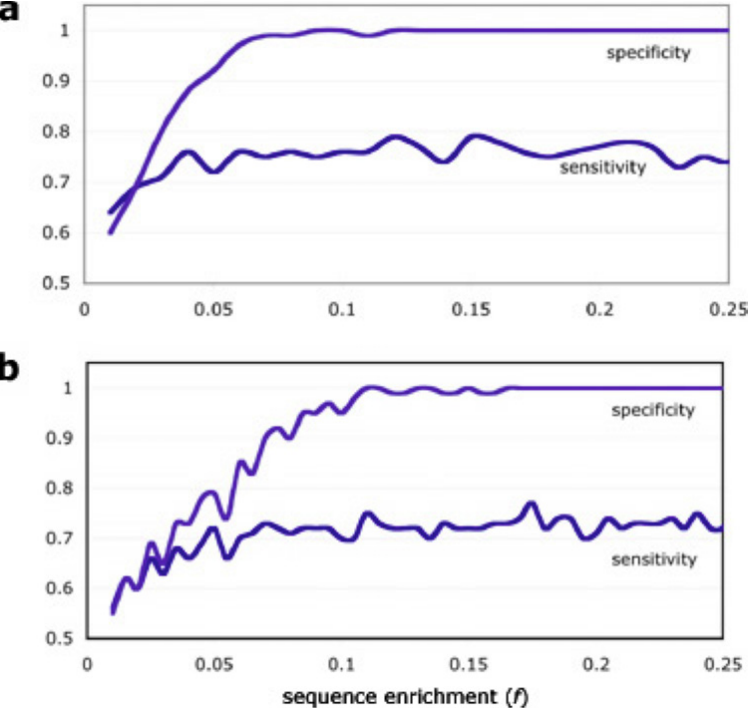
**Detection of exposure to representative specificities**. Representative plots of sensitivity and specificity for detecting exposure at various levels (*f*; see Methods for details): (a) *Streptococcus pneumoniae *serotype 6B and (b) *S. pneumoniae *ser. 26F. In general sensitivities reached between 0.7 and 0.8 when repertoires were enriched to at least 5 percent (*f *= 0.05), and specificities reached between 0.95 and 1.

A training collection for each specificity was assembled comprising 10 exposed and 10 unexposed sets. An additional 50 test sets, whose exposed/unexposed status was known to us but not to the algorithm, were presented for assignment. Performance was measured by sensitivity and specificity (see Methods). Figure 3 shows results for two typical simulations. Sensitivity generally reached between 0.7 and 0.8 when exposure-specific antibodies/sequences were five percent of the total; specificity was higher (most likely due to false negatives in the sensitivity because of the small size of the reference sets). Sensitivity was improved by increasing the size of and enrichment in the sets in the reference collection. (Here the terms "sensitivity" and "specificity" are used in the epidemiological sense; see Methods.)

## Discussion

The majority of modern clinical tests assay for just one analyte at a time [18]. They determine the presence or absence of the analyte, and sometimes its quantity, but provide no information about other analytes. For example, a nucleic acid test for HIV-1 determines whether or not HIV-1 RNA is present in blood, and how much, but provides no information about, for example, the presence of antibodies to CMV. Although such tests are the mainstay of modern medicine, conceptually, they are limited to providing a "20 questions," yes-or-no approach to diagnosis.

The major exception is the standard culture-based method for diagnosing bacterial infections. In this method, the first step is to apply a clinical sample to standard culture media to see what grows [19]. This method is powerful in that it presupposes little about the identity of the bacteria: it can distinguish among many bacteria with a single test, and often reveals the presence of species that were clinically unexpected. Conceptually, this is an open-ended, "what-is-there" approach to diagnosis. It is of general interest in medicine to develop more diagnostic techniques that use this approach.

Antibodies play a crucial role in protective immunity and immunopathology, and also are important in surveillance against cancer [1]. The relationship between antibody gene sequence and epitope specificity is complex, but several studies have shown that certain gene segments and gene segment combinations are used preferentially against specific epitopes, antigens, or sets of antigens – what we here call "specificities" [7,10-14]. The identity and frequency of gene segments or combinations define antibody repertoires.

In this paper we have analyzed the growing, albeit limited, data that exists on VDJ_*H *_combination defined repertoires to see whether they might one day provide an open-ended diagnostic for antigens to which a person has been exposed. For statistical confidence, we analyzed only those specificities for which at least eight antibodies have been sequenced and annotated for V_*H*_, D, and J_*H *_gene segment use. A similar amount of systematic data for immunoglobulin light chains and T cell receptors is still unavailable, and so the present analysis was limited to immunoglobulin heavy chains.

Our data set represented nearly every gene segment family, and at frequencies similar to those seen in two healthy individuals in a previous study [15]. One interpretation is that this reflects an intrinsic bias in the frequency with which different VDJ_*H *_combinations are formed or expressed. Another interpretation is that the specificities in our data set are representative of the exposures that shape repertoires in healthy individuals, since certain types of antigens – bacterial polysaccharides, for instance – select for certain canonical structures in antibodies, and segments of the same gene family are more likely to produce similar structures [16]. These two interpretations are not mutually exclusive.

The narrowness or breadth of the repertoires for individual specificities (Fig. 2) could simply reflect the number of epitopes per specificity. For example, the antibodies against factor VIII, which formed a narrow repertoire, are known to have been raised against relatively well defined domains of factor VIII that comprise few epitopes [20], while antibodies against dsDNA, which formed a broad repertoire, were not raised this way [21].

The fact that the same VDJ_*H *_combinations were recovered from multiple individuals in many repertoires (e.g., the *S. pneumoniae *PS repertoires [10,11]) suggests that despite genetic differences, different individuals may often use the same or at least overlapping sets of VDJ_*H *_combinations in the antibodies they make against a given epitope. These could be called "public" or "semi-public" combinations [7,3]. Such commonalities might shed light on the evolutionary forces – repeat exposure to particular infectious agents, for example [4] – that may have shaped and maintained germline gene segment diversity. Further sequencing experiments using specificities defined at the epitope level would be useful to determine how often and to what epitopes public and semi-public combinations occur. The more frequent public combinations turn out to be, the more narrowly defined specificities can be and remain detectable, and vice versa.

Repertoires' VDJ_*H *_combinations overlapped rarely (Fig. 3b), and less often than would be predicted by chance (*p *≤ 0.011). Specifically, for any two specificities chosen at random, chances were 98.3 percent that they overlapped by 10 percent or less. This suggests that determining VDJ_*H *_usage for a sampling of antibodies can be used to identify exposure to a particular antigen or set of antigens with reasonable specificity.

To further explore this idea, we conducted a set of simulation experiments to see whether individuals could one day be diagnosed as being exposed or not exposed to a given specificity (relative to a normal baseline) by assaying for enrichment of certain VDJ_*H *_combinations. We show that even at modest levels of enrichment, which represents an increased frequency of B cells specific to a certain exposure, and using just 10 reference sets as the "gold standard" for exposure, assignment of unknown sets as either exposed or unexposed was possible with a high degree of sensitivity and specificity. In principle, such a sequence-based method has the advantage of being able to detect patterns of exposure even when the specificity of the antibodies or the identity of the offending agent is completely unknown. This "open-ended" approach is most useful for the early detection of emerging diseases, and will become practicable as improvements in sequencing technology make it possible to use in the clinic [22]. Data on antibody titers and functionality will doubtless add to the utility of this approach.

## Conclusion

In sum, this study is the first to our knowledge that investigates the relationship between antibody specificity and VDJ_*H *_segment usage for a large number of sequenced antibodies. Further sequencing studies should make it possible to refine the conclusions presented here, and also to assess the contribution of light chain in antibodies and of alpha and beta chains in T cell receptors to antigen specificity in human immune responses. Whether or not large-scale sequencing will prove useful as a future diagnostic tool will depend on these further studies.

## Methods

### Antibody repertoire data

The ImMunoGeneTics database (IMGT; ) is a publically available curated online repository of ~88,000 sequenced immunoglobulin and T cell receptor genes from a number of species [2]. We extracted all ~531 entries that contained recombined human immunoglobulin genes annotated with V_*H*_, D, and J_*H *_gene segment use and antigen specificity.

To approximate only natural repertoires, we limited our analysis to sequences isolated from B cells of individuals, and excluded all sequences that had been designed or modified *in vitro*. Allowed sequences included ones obtained from Epstein-Barr Virus (EBV)-immortalized B cells, through combinatorial cloning or phage-display libraries constructed from B cells of antigen-exposed patients, and from single sequenced B cells. For statistical power we considered only those specificities that had at least eight sequences in IMGT. There were 16 such specificities, comprising a total of 292 individual antibody sequences (mean, 18 sequences per specificity; range, 8–41).

### Frequency distributions and overlap

We calculated and tabulated V_*H*_, D, and J_*H *_frequency distributions from all specificities and calculated their pairwise overlap computationally. Because specificities generally differed in the number of unique VDJ_*H *_combinations in their repertoires, overlap was not symmetric: for example, if one specificity's repertoire comprised five different VDJ_*H *_combinations, and another specificity's repertoire had those same five combinations as well as an additional 15, the overlap would be 100 percent in one direction, but only 25 percent in the other.

Student's t-test for two independent samples was used to obtain p-values for calculated vs. observed frequencies of VDJ_*H *_combinations for each repertoire. Heatmap plots were made using R .

Humans most commonly encode 38 functional V_*H *_genes, 23 functional D genes, and 6 functional J_*H *_genes, as well as a number of pseudogenes [2]. These allow for a theoretical maximum of 38 × 23 × 6 = 5,244 possible VDJ_*H *_combinations. In addition, many Caucasians contain a partial duplication of the V_*H *_region that results in 46 functional V_*H *_genes [2]; this partial duplication allows for a theoretical maximum of 46 × 23 × 6 = 6,348 VDJ_*H *_combinations. For a person with a maximum of 5,244 possible VDJ_*H *_combinations, the probability that two sets of 10 randomly chosen combinations will not overlap at all is approximately [(5,244 - 10)/5,244]^10 ^= 0.98, or 98 percent. The probability that a third set of, for example, seven combinations will not overlap at all with either of these two sets is approximately [(5,244 - 10)/5,244]^10 ^× [(5,244 - 10 - 10)/5,244]^7 ^= 0.96, or 96 percent. The probability of overlap among any group of sets may be approximated by extending this method.

### Simulations

We built a pattern-detecting computer algorithm for detecting enrichment of antibody sequences that correspond to particular specificities [23] (single-hidden layer, feed-forward neural networks with backpropagation; Brainstem v1.4; ).

For each specificity, the algorithm was trained on a reference collection representing 10 exposed and 10 unexposed sequence sets as follows. Each set constituted a list of the frequency of each of 100 VDJ_*H *_combinations drawn equally from all the specificities in the data set. For each specificity, we sampled a fraction (*f*) of combinations from that specificity's repertoire according to their frequency distribution, allowing resampling. The remainder were sampled from all sequences in the data set, including those of the chosen repertoire, again allowing resampling. This remainder represents a background of noise against which a signal – enrichment of specific sequences – might be detected. 0 <*f *≤ 1 for exposed sets and *f *= 0 for unexposed sets. Hence, a set is "exposed" if it is statistically enriched for sequences a particular specificity, and "unexposed" otherwise. Note that unexposed sets will contain some sequences from the chosen exposed repertoire by chance, just at lower frequency than in exposed sets. Our question was, how well can we assign, or "diagnose," exposure: i.e., how well can we detect enrichment.

The algorithm was used to evaluate test sets, each comprising an additional 25 exposed and 25 unexposed patients. For each specificity, the algorithm was trained at 0.01 ≤ *f *≤ 0.025 (for the exposed patients) and tested over the range 0.01 ≤ *f *≤ 1. To quantify the results, we calculated the sensitivity [(true positives)/(true positives + false negatives)] and specificity [(true negatives)/(true negatives + false positives)] of the algorithm for each test set. These are standard metrics for diagnostic tests in the clinical setting [18].

## List of abbreviations

dsDNA, double-stranded DNA; HIV-1, human immunodeficiency virus type 1; Sp, *Streptococcus pneumoniae *serotype; fVIII, clotting factor VIII; HBsAg, hepatitis B virus surface antigen; IIb/IIIa, glycoprotein IIb/IIIa; MAG, myelin-associated glycoprotein; PL, phospholipid; TPO, thyroid per-oxidase; CMV, cytomegalovirus.

## Authors' contributions

R.A.A. performed all the work presented in this paper.

## Supplementary Material

Additional File 1 VDJtable.pdf, is a PDF file that contains a table listing VDJ combinations for all specificities analyzed in this paper. Click here for file
