# Supplementary material for: Specificity and overlap in gene segment-defined antibody repertoires
Source: BMC Genomics. 2005 Oct 28;6:148. doi: 10.1186/1471-2164-6-148 (PMC1277825; doi:10.1186/1471-2164-6-148)
Supplement: Additional File 1 — VDJtable.pdf, is a PDF file that contains a table listing VDJ combinations for all specificities analyzed in this paper. [file 1471-2164-6-148-S1.pdf]

Supporting Table 1: VDJ_H repertoires for all specificities

specificity	observed VDJ_H combinations
HBsAg (HBV)	V1-46/D3-22/J5, V1-69/D3-9/J6, V3-20/D3-3/J6, V3-21/D1-26/J5, V3-23/D1-26/J4, V3-23/D6-13/J4, V3-30/D3-9/J4, V3-30/D5-5/J4 (2), V3-49/D5-12/J4, V4-39/D3-10/J6, V4-4/D3-10/J4
Fab (human)	V1-2/D3-10/J6, V3-15/D3-10/J4, V3-23/D5-5/J4, V3-48/D3-16/J6, V3-7/D6-25/J4, V3-73/D3-10/J6 (2), V3-74/D1-26/J4, V4-39/D1-1/J6, V5-51/D1-26/J5, V5-51/D3-9/J4
gp120 (HIV)	V1-24/D1-26/J4, V1-69/D1-14/J6, V1-69/D1-7/J6, V1-69/D2-2/J4, V1-69/D2-2/J6, V1-69/D2-8/J5 (2), V1-69/D3-22/J4, V1-69/D3-3/J6 (2), V1-69/D3-9/J2, V1-69/D4-17/J6, V1-69/D4-23/J3, V1-69/D4-4/J2, V1-69/D5-12/J1, V1-69/D6-19/J4, V1-f/D1-26/J3, V1-f/D1-7/J4, V1-f/D2-15/J4, V2-5/D3-3/J5, V3-23/D3-3/J4, V3-30-3/D3-3/J3, V3-64/D3-22/J3, V4-31/D3-3/J3, V4-39/D5-5/J4, V4-4/D6-6/J5
gpIIb/IIIa (human)	V1-2/D3-10/J6, V1-2/D5-5/J4, V1-69/D2-2/J4, V3-20/D2-2/J3, V3-21/D1-14/J3, V3-23/D3-10/J4, V3-30/D3-22/J4, V3-30-3/D3-16/J4, V3-49/D1-14/J6, V4-39/D4-4/J4, V4-4/D3-22/J3, V5-51/D1-7/J3, V5-51/D2-21/J4, V6-1/D6-19/J4
PS (*S. pneumo* 6B)	V3-15/D3-3/J4, V3-15/D5-5/J4, V3-23/D2-2/J1 (3), V3-23/D3-9/J4 (5), V3-23/D4-23/J1, V3-23/D6-19/J4 (2), V3-30/D2-2/J4 (3), V3-7/D1-26/J4 (4), V3-7/D1-7/J4 (5), V3-7/D3-16/J4 (15), V3-73/D3-10/J6
TPO (human)	V1-2/D3-9/J6 (2), V1-3/D1-26/J6 (2), V1-3/D2-8/J4, V1-3/D3-10/J3, V1-3/D3-10/J4 (2), V1-3/D4-17/J4 (2)V1-3/D4-4/J4 (3), V1-3/D5-24/J6, V1-69/D3-10/J6 (8), V1-69/D4-17/J5, V1-8/D1-26/J6, V3-21/D1-1/J5 (2), V3-21/D3-16/J5, V3-21/D3-9/J5, V3-21/D5-12/J5 (12), V5-51/D5-5/J6

Supporting Table 1: VDJ_H repertoires for all specificities, continued

specificity	VDJ_H combination
MAG (human)	V1-8/D6-19/J6, V3-15/D1-26/J5, V3-15/D3-10/J3, V3-15/D4-17/J4, V3-48/D3-22/J4, V3-9/D6-19/J3, V4-4/D3-3/J5, V4-61/D3-3/J3, V4-b/D2-2/J5
cardiolipin (human)	V1-2/D2-15/J4, V1-2/D5-12/J4, V1-3/D3-3/J4, V1-69/D3-16/J3, V1-69/D3-3/J4, V2-5/D3-3/J5 (2), V3-21/D5-5/J4 (2), V3-48/D1-26/J5, V3-48/D6-19/J4, V4-b/D5-5/J4
RhD (human)	V1-2/D1-26/J3, V1-2/D2-15/J4, V1-2/D3-16/J6, V1-2/D3-22/J3, V1-69/D6-13/J6, V2-26/D4-4/J6 (2), V3-21/D3-9/J6, V3-30/D3-10/J6 (2), V3-30/D3-22/J3, V3-30-3/D2-21/J6, V3-33/D2-21/J6, V3-33/D3-22/J6, V3-33/D3-3/J6, V3-33/D4-17/J4, V3-33/D4-23/J6, V3-33/D6-19/J5, V4-34/D3-10/J4, V4-34/D3-16/J4, V4-34/D6-6/J6, V4-39/D6-13/J2
factor VIII (human)	V1-18/D3-3/J6 (3), V1-24/D2-2/J4 (2), V1-24/D3-3/J3, V1-69/D3-10/J6 (10), V1-69/D3-9/J3 (2), V1-69/D6-13/J3
PS (*S. pneumo* 23F)	V3-23/D1-1/J4 (2), V3-23/D1-1/J5, V3-23/D1-26/J5, V3-23/D3-3/J6, V3-23/D5-5/J4 (2), V3-30/D3-10/J4, V3-30/D5-24/J4 (2), V3-30/D6-19/J4 (2), V3-33/D6-25/J4, V3-48/D6-13/J2 (4), V3-64/D6-6/J6 (4), V3-7/D3-10/J4, V4-59/D3-10/J5
E. histolytica	V1-18/D4-4/J4, V1-46/D4-23/J4, V3-21/D2-15/J6, V3-30/D2-2/J6 (3), V3-48/D2-2/J4, V3-53/D6-6/J4, V3-64/D2-15/J4

Supporting Table 1: VDJ_H repertoires for all specificities, continued

specificity	VDJ_H combination
DNA (human)	V1-46/D2-2/J3, V1-46/D3-16/J3, V1-46/D3-9/J4, V1-69/D4-17/J3, V1-8/D1-1/J5, V2-5/D6-19/J4, V3-30/D6-6/J4, V3-33/D3-9/J3, V3-33/D3-9/J4, V3-33/D5-5/J3, V3-48/D2-2/J5, V3-7/D1-1/J4, V3-7/D2-15/J4 (2), V3-9/D1-26/J5, V3-9/D1-7/J3, V3-9/D2-15/J4, V3-9/D2-21/J5, V4-31/D5-5/J5, V4-34/D1-1/J6, V4-59/D6-13/J6 (2)
PL (human)	V1-2/D3-9/J5, V1-3/D3-10/J4, V3-23/D3-10/J4, V3-74/D2-21/JH4, V4-31/D3-3/J4, V4-34/D2-2/J6, V4-39/D1-26/J4, V4-39/D5-24/J4, V6-1/D6-13/J4
dsDNA (human)	V3-23/D1-1/J2, V3-23/D4-17/J4, V3-30/D3-22/J3, V3-64/D4-17/J5, V3-66/D2-2/J3, V3-74/D3-22/J3, V4-59/D2-21/J4, V5-51/D4-23/J5
myosin (human)	V1-2/D2-15/J5, V1-2/D6-13/J3, V1-46/D2-2/J4, V1-69/D6-13/J2, V2-5/D7-27/J4, V3-15/D3-22/J4, V3-30/D2-2/J4, V3-30/D3-16/J4, V3-43/D5-24/J3, V3-48/D1-26/J3, V3-53/D3-10/J4, V3-9/D3-10/J1, V4-59/D1-7/J4, V6-1/D1-7/J3

Numbers in parentheses () indicate the multiplicity of sequences that used this VDJ_H combination.
